# Late Recurrence of Renal Cell Carcinoma in Unusual Sites 23 Years After Nephrectomy

**DOI:** 10.7759/cureus.45707

**Published:** 2023-09-21

**Authors:** Efrain Castillo, Kassandra Martinez

**Affiliations:** 1 Medicine, Universidad Latina de Panama, Panama City, PAN; 2 Internal Medicine, Hospital Santo Tomás, Panama City, PAN

**Keywords:** clear renal cell carcinoma, late recurrence, pancreatic metastasis, metastasis, renal cell carcinoma

## Abstract

Renal cell carcinoma (RCC) is a common malignancy in the elderly population and it is notorious for its mechanism of late metastasis to unusual anatomical sites. Late recurrences are common following curative treatment, such as radical nephrectomy. Pancreatic metastases and hepatic metastatic lesions make the diagnosis and classification of a primary tumor challenging. This necessitates a high index of suspicion and an extensive interrogation.

We present the case of a 68-year-old Hispanic female with progressive pain in the right upper quadrant, weight loss, and decreased appetite. She has a history of renal cell carcinoma treated with radical nephrectomy and radiotherapy. A pancreatic biopsy was performed based on the ultrasound and computed tomography findings at a private clinic. Subsequently, the patient was referred to the Instituto Oncologico Nacional (ION) due to immunohistochemical features suggestive of a well-differentiated neuroendocrine tumor. At ION, a more comprehensive medical history was obtained, and the immunohistochemistry panel was extended, confirming the diagnosis of late recurrence of RCC. One possible explanation for the very late relapse of our patient is the presence of malignant cells that were spared or seeded during the surgical intervention and remained dormant or latent, subsequently spreading via hematogenous dissemination or via the lymphatic system. We highlight the importance of medical history, pathological examination, and immunohistochemical analysis in establishing a differential diagnosis, given the nature of RCC, which can often present asymptomatically and has a propensity for late recurrence. Further research should focus on developing standardized surveillance protocols for such cases.

## Introduction

Renal cell carcinoma (RCC) is a common malignancy in the elderly population that originates from the renal cortex and the renal tubular epithelium. RCC accounts for approximately 2% of all cancer diagnoses and deaths and higher incidence rates in developed countries [1]. RCC is characterized by its strong tendency to metastasize in unpredictable patterns and high rates of recurrence post-resection [2]. In most cases, it occurs sporadically with risk factors such as smoking, obesity, a history of sickle cell anemia, nephrolithiasis, and occupational exposure [3]. Most patients are asymptomatic. The classical triad of flank pain, hematuria, and a palpable abdominal renal mass is seen in 15% of cases [4]. RCC is generally resistant to chemotherapy drugs and radiotherapy, so nephrectomy is the first-line treatment for the non-metastatic disease [5]. Leukemoid reaction, hypercalcemia, and hypertension are common complications [6].

## Case presentation

A 68-year-old Hispanic female with a history of right chromophobe cell RCC treated with open radical nephrectomy and adjuvant radiotherapy for 10 years presented to the emergency department with progressive pain localized to the right hypochondrium and radiated towards the back, involuntary twenty-pound weight loss, and generalized weakness. About five months ago, the patient developed a non-tender and generalized abdominal discomfort over the course of two months treated with dietary modifications and oral analgesics at a primary care clinic. 

Her family history was remarkable for osteosarcoma in her sister, small cell lung carcinoma in her grandmother, and colorectal cancer in her grandfather, but no family history of renal cancer. The patient's medication history includes daily metformin, rosuvastatin, and lisinopril. She reported occasional non-steroidal anti-inflammatory drugs (NSAIDs), and denied alcohol consumption or illicit drug use. The patient reported a smoking history of 7.5 pack-years. 

On physical examination, dry mucous membranes, and a pale and cachectic appearance are noted. Abdominal examination showed a superficial scar from the previous nephrectomy, right upper quadrant abdominal tenderness without peritoneal irritation signs, normal bowel sounds, the liver was palpated 3 cm below the costal margin, and the spleen was not palpable. Vital signs revealed blood pressure of 150/90 mm Hg, a low-grade fever (37.7º Celsius), a BMI of 21.5 kg/m^2^, a heart rate of 70 beats per minute, and a respiratory rate of 16 per minute. Past medical history is relevant for type 2 diabetes mellitus.

Laboratory tests showed normocytic normochromic anemia, elevated lactate dehydrogenase (325 U/I), hypercalcemia (11.5 mg/dL), hypoalbuminemia (3.4 g/dL), increased erythrocyte sedimentation rate (65 mm/1st hour, 100/2nd hour) (Table 1).

**Table 1:** Main laboratory data on admission.

Lab tests	Value	Reference range
WBC count, ×10^3^/μL	5.4	4.5–11
Hb, g/dL	10.8	Male: 13.5-17.5; female: 12.0-16.0
Platelet count, x10^9^/L	220	150-400
TSH μU/mL	3.6	0.4–4.0
T3, ng/dL	138	100-200
T4 µg/dL	7.1	5-12
Glucose, mg/dL	55	Fasting: 70–100
LDH, mg/dL	325	45-200 U/L
Serum calcium	11.5	8.4-10.2 mg/dL
Albumin, g/dL	3.3	3.5-5.5
ESR, mm/hr	65	Male: 0–15; female: 0–20.
BUN, mg/dL	21	7–18
SCr, mg/dL	1.41	0.6–1.2
ALT, U/L	54	10–40

**Figure 1:**
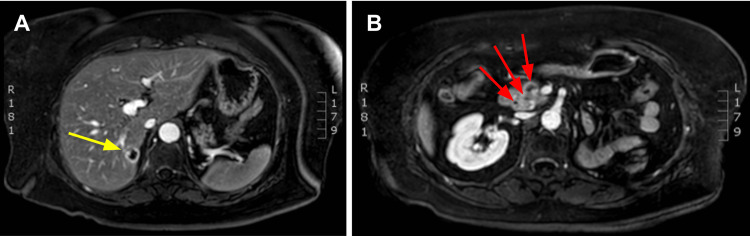
Contrast-enhanced abdominal MRI. (A) Focal liver lesion of 1.7 cm in segment 6th of the right hepatic lobe (yellow arrow). (B) Multiple nodular lesions in the pancreas (red arrows).

A hepatobiliary ultrasound revealed multiple hypoechoic nodular lesions in the pancreas and a single lesion in the left hepatic lobule. On computed tomography of the abdomen and pelvis (CTAP), a focal liver lesion of 1.7 cm in the 6th segment of the right hepatic lobe and multiple nodular lesions in the pancreas are evidenced and confirmed with abdominal magnetic resonance imaging (MRI) (Figure 1). No signs of lymphadenopathy are present. The rest of the CTAP was unremarkable.

A fine-needle pancreatic biopsy was performed, showing multiple necrotized nodules. On pathology examination, the mass is necrotic, grey in color, hard consistency. Resected regional lymph nodes were negative for malignancy. No KRAS mutations were identified on cytologic analysis. At the first clinic, immunohistochemical (IHC) staining studies were conducted and tumor cells showed immunoreactivity with KI-67, CD56, CK19, and CKAE1/AE3. The patient is referred to the ION with suspected solid pseudopapillary neoplasm, acinar cell carcinoma (ACC), and hepatic metastases.

During admission to the Oncology Center, an extensive interrogatory was conducted and a history of RCC was found. As a result, the decision to expand the initial IHC panel was made. Tumor cells revealed positivity for the cluster of differentiation 10 (CD10), cytokeratin 7 (CK7), vimentin, PAX-8, cyclin D1, RCC, pankeratin and a cytoplasmatic expression of β-catenin. Cells also revealed negative results for cytokeratin 20 (CK20), CK-19, CK7, and MART-1 staining (Tables 2-3). 

**Table 2:** Immunohistochemical panel from the first clinic.

Marker	Results
Chromogranin A	-
Synaptophysin	-
KI-67	+
CD-56	+
CK-19	-
CKAE1/AE3	+

**Table 3:** Immunohistochemical panel findings from the second clinic.

Marker	Results
PAX-8	+
CD10	+
Cyclin D1	+
Pankeratin	+
Vimentin	+
RCC	+
CK-19	-
CK7	-
MART-1	-
β-catenin	Cytoplasmatic

**Figure 2:**
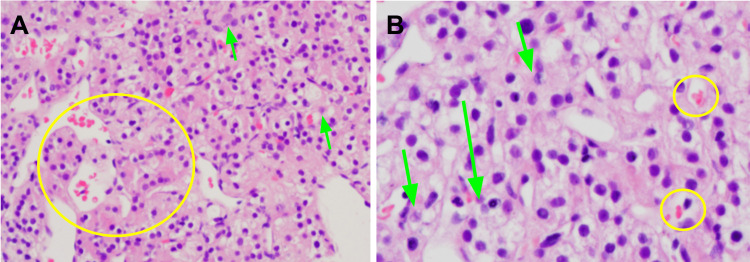
Histopathological evaluation of the pancreatic tissue. (A) Round cell sheets with slight atypia and clear cytoplasm (green arrows) with a delicate capillary network (yellow circles); H&E 20X. (B) H&E 40X.

**Figure 3:**
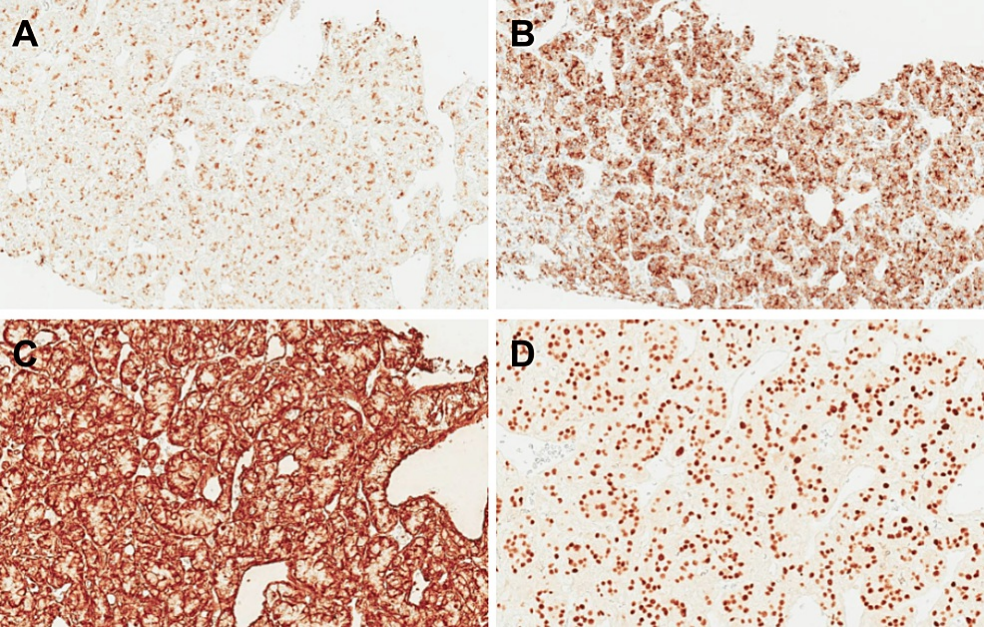
Immunohistochemical features of the excised specimen. (A) Renal cell carcinoma (RCC) showed a positive nuclear expression of 30%, (B) CD10 with a positive nuclear expression of 90% of the cytoplasmic tissue, (C) Vimentin showed a positive nuclear expression of 100% of the membranous tissue and (D) Pax 8 showed positive nuclear expression of 100% of the cytoplasmic tissue.

On microscopy, sections of tissue samples sections revealed the proliferation of solid and gland-like formations of polygonal cells, round cell sheets with clear cytoplasm and slight atypia, sites of scattered necrosis with a delicate capillary network resembling metastases of RCC, versus the initially suspected neuroendocrine tumor (Figure 2). The second IHC panel confirmed the diagnosis (Figure 3). 

**Figure 4:**
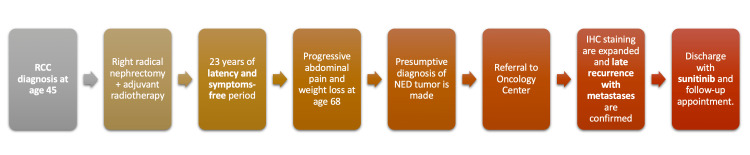
Timeline and description of events of our patient. RCC: renal cell carcinoma; NED: neuroendocrine; IHC: immunohistochemical.

The patient was admitted to the Oncology Department and was started on sunitinib (Sutent®) 50 mg, one tablet per day for one month initially, and a follow-up visit was scheduled for three months to monitor treatment response. Figure 4 summarizes the timeline of events. Late relapse of RCC with hepatic and pancreatic metastases is confirmed, ruling out NED neoplasia and acinar cell carcinoma (ACC) as the main diagnostic suspicions in previous institutions. The patient was managed with sunitinib, which resulted in a partial response for 18 months, and was later changed to axitinib due to tumor progression.

Our patient’s case is consistent with a rare and late recurrence of RCC presenting in the liver and pancreas, despite a presumed definitive nephrectomy over two decades prior. The patient remains asymptomatic and in complete remission to this day.

## Discussion

We have reported an interesting case of a late and initially missed recurrence of renal cell carcinoma presenting in the liver and pancreas, these represent a rare anatomical site of metastasis. We aim to raise awareness and highlight the importance of an extensive interrogatory in cases of a past medical history of RCC due to the unknown mechanism of metastases and dissemination to rare sites that present asymptomatically. Current literature describes that the extent and histopathology of the tumor determine a patient's prognosis [7]. Usually, in the early stages, the growth that is limited to the kidney tends to represent a good prognosis for which targeted therapies prolong survival rates. Currently, with radiological advances, patients with RCC and a localized disease are diagnosed incidentally. The classic RCC triad of flank pain, hematuria, and a palpable abdominal renal mass is present in less than 15% of patients and is associated with locally advanced disease [8]. 

Patients with RCC can present with or subsequently develop systemic symptoms or paraneoplastic syndromes [9]. In some cases, these syndromes arise due to ectopic production of hormones such as erythropoietin, parathyroid hormone-related protein, gonadotropins, human placental lactogen, an adrenocorticotropic hormone-like substance, and renin. Commonly described presentations are anemia, hypercalcemia, erythrocytosis, thrombocytosis, cachexia, secondary amyloidosis, fever, hypertension, hepatic dysfunction, or Stauffer's syndrome, among others [10]. 

Most patients who are treated with nephrectomy for renal cell carcinoma for a clinical T1-T3 N0 disease achieve a complete response; however, 20% to 30% of patients will recur [11]. Remarkably, RCC is notorious for its capacity to recur, even after many years from a curative nephrectomy. The risk of relapse is higher within the first five years of diagnosis, with approximately 25% of patients with distant metastases or advanced locoregional disease at diagnosis [12]. Recurrences that occur at least 10 years after radical nephrectomy of the primary tumor are late relapses, but the risk of recurrence in these patients is higher after five years of the nephrectomy [13]. Metastases can invade adjacent organs by contiguity or disseminate to distant sites via hematogenous or lymphatic spread. RCC metastasizing to the pancreas is uncommon and develops in almost 3% of patients with metastatic RCC [14]. However, of those detected in clinical practice, the majority arise from RCC. Our report represents the case of a very late recurrence since the current literature describes the median time from nephrectomy to recurrence of 8.3 years [15]. As of today, there is no report of a very late recurrence of RCC in the liver and pancreas concurrently.

Hepatic metastatic lesions are more common than pancreatic metastases, with the liver being affected in 18% of cases [16], and pancreatic lesions with an estimated incidence of 3%-10% [17]. In our case, the single liver lesion and the multiple pancreatic nodular lesions necessitated an extensive evaluation to differentiate a neuroendocrine tumor and primary liver cancer from metastatic cancer. While neoplasms arising from the liver show are positive for HepPar1, villin, CD10, CK8/CK18, and arginase-1, tumors originating from adrenocortical glands are positive for steroidogenic factor 1 (SF1), vimentin, calretinin, inhibin, MelanA/MART1, and synaptophysin. However, RCC demonstrates positivity for epithelial markers, including RCC, CD10, and PAX8, but negative results for CK7, CK20, HepPar-1, or SF1 [18]. The results received from the second IHC studies confirmed the diagnosis with positive RCC, CD10, PAX8, and cytoplasmatic β-catenin expression with a past medical history of RCC. 

Late recurrences in RCC pose a challenge in oncology, often occurring more than a decade after curative treatments. These late recurrences are believed to involve the phenomenon of tumoral dormancy or latency. Several mechanisms are attributed, including the malignant cells' intrinsic ability to inactivate and evade immune response long-term by the slowing down of the cell’s proliferative phase and increase in the quiescent phase, the constant and uncontrolled growth of metastatic cells under these conditions. The decline in immune function is thought to precipitate the reactivation of latent lesions, causing metastasis [19]. Also, the angiogenic switch plays a pivotal role in maintaining tumoral growth by increasing endothelial cell proliferation and tumor growth [20]. The tumor microenvironment and its complex cellular interactions can influence dormancy or reactivation, of cancer stem cells, and genetic and molecular factors are also hypothesized. Extracellular tumor-associated microvesicles are thought to contribute to immune evasion and affect drug response for RCC by spreading via hematogenous routes and seeding any distant organ [21]. 

Currently, medical literature lacks comprehensive clinical, genetic, and histological data, making it imperative for further research to be conducted. This is important in order to establish standardized protocols for the management of metastases, ultimately improving patient outcomes and long-term survival. Future research should focus on long-term surveillance methods, given the unpredictable nature of RCC.

## Conclusions

We report a case of RCC with late relapse in the liver and pancreas 23 years after radical nephrectomy. Hence, latent RCC metastasis may develop due to the decline in immune function with aging, a phenomenon described as "tumor dormancy". A history of RCC warrants careful attention to evaluating for recurrence regardless of the number of years after surgery, whether the condition is hepatic or pancreatic, both of which are rare sites of metastasis. This case highlights the importance of anamnesis as an irreplaceable and crucial tool to guide imaging, pathology, and immunohistochemical studies in the diagnostic approach for cancer patients. It also emphasizes the necessity of involving a multidisciplinary team to reach a definitive diagnosis. Regardless of the provisional differential diagnosis made and the number of years after the nephrectomy, the medical evaluation must assess past medical history and clinicians must rule out recurrences.
